# Cell–cell junctional mechanotransduction in endothelial remodeling

**DOI:** 10.1007/s00018-016-2325-8

**Published:** 2016-08-09

**Authors:** Yvonne L. Dorland, Stephan Huveneers

**Affiliations:** 1 Department of Molecular Cell Biology, Sanquin Research and Landsteiner Laboratory, University of Amsterdam, Amsterdam, The Netherlands; 2 Department of Medical Biochemistry, Academic Medical Center, University of Amsterdam, Amsterdam, The Netherlands

**Keywords:** Adherens junction, Mechanosensing, Catenin, PECAM-1, Cytoskeleton, Endothelial integrity, Permeability, Vascular stiffness, Cardiovascular disease

## Abstract

The vasculature is one of the most dynamic tissues that encounter numerous mechanical cues derived from pulsatile blood flow, blood pressure, activity of smooth muscle cells in the vessel wall, and transmigration of immune cells. The inner layer of blood and lymphatic vessels is covered by the endothelium, a monolayer of cells which separates blood from tissue, an important function that it fulfills even under the dynamic circumstances of the vascular microenvironment. In addition, remodeling of the endothelial barrier during angiogenesis and trafficking of immune cells is achieved by specific modulation of cell–cell adhesion structures between the endothelial cells. In recent years, there have been many new discoveries in the field of cellular mechanotransduction which controls the formation and destabilization of the vascular barrier. Force-induced adaptation at endothelial cell–cell adhesion structures is a crucial node in these processes that challenge the vascular barrier. One of the key examples of a force-induced molecular event is the recruitment of vinculin to the VE-cadherin complex upon pulling forces at cell–cell junctions. Here, we highlight recent advances in the current understanding of mechanotransduction responses at, and derived from, endothelial cell–cell junctions. We further discuss their importance for vascular barrier function and remodeling in development, inflammation, and vascular disease.

## Introduction

The inner lining of blood vessels consists of a monolayer of specialized cells called the vascular endothelium. High coherence between the endothelial cells enables a controllable barrier for blood components and inflammatory cells between circulation and tissues. However, being part of a highly dynamic tissue itself, the endothelium is constantly subject to changes in mechanical forces. This accounts for endothelial cells in developing vasculature during embryogenesis, but also for the endothelium in existing vessels in adults, which experience forces that derive from pulsatile blood flow, vessel wall contractions, and trafficking of immune cells. The endothelial monolayer has the fascinating capability to adapt accordingly to all these mechanical inputs while maintaining its crucial vascular barrier function. However, well-adjusted endothelial responses to forces are challenged by stiffening of the vascular wall upon aging [1, 2]. Failure of the endothelial monolayer to adapt to changes in the magnitude or direction of forces has direct consequences on vascular permeability, and is, therefore, regarded as an important cause of vascular diseases, such as acute edema, chronic inflammation, hypertension, and atherosclerosis [3]. Cells convert mechanical information into biological responses via so-called mechanotransduction processes. Increasing our understanding of vascular mechanotransduction pathways may yield potential targets or new approaches to restore barrier function in these vascular diseases.

Currently, much vascular cell research focuses on identifying molecular events that may explain how the endothelium senses and responds to mechanical cues. Coupling between extracellular environment and cellular interior occurs via multiprotein transmembrane complexes that are based on integrins, cadherins, mechanosensitive ion channels, G-protein-coupled receptors, and receptor tyrosine kinases [4–6]. In addition, upon alterations in experienced force, dedicated mechanotransduction complexes undergo structural deformations [7]. Such mechanically induced conformational changes can determine association or dissociation of specific proteins by controlling the exposure of protein-binding domains. Multiple mechanotransduction-related events in various tissues have been extensively reviewed in [7–15]. In this review, we highlight recent discoveries in endothelial mechanotransduction pathways that regulate, or are regulated by, endothelial cell–cell junctions and we will discuss the vascular processes they likely associate with.

## Mechanical regulation of endothelial cell–cell junctions

Endothelial monolayer integrity is maintained by VE-cadherin-based adherens junctions, an essential multiprotein cell–cell adhesion structure, which consists of the transmembrane receptor VE-cadherin, intracellularly associated catenins, and other regulatory proteins [16]. The endothelial adherens junctions are formed in conjunction with other cell–cell adhesions based on receptors, such as nectins, claudins, occludins, JAMs, and PECAM-1. A mechanotransduction role for VE-cadherin initially became apparent from fluid flow studies, in which VE-cadherin, in combination with VEGFR2 and PECAM-1, turned out to be required for endothelial cell alignment in the direction of fluid flow [17]. A second important observation was made while studying the remodeling of endothelial adherens junctions using traction force microscopy. These experiments show that augmented cytoskeletal-pulling forces on VE-cadherin-based cell–cell junctions increase junctional size without a loss of tension on the junction itself [18]. This suggests that the VE-cadherin complex responds to increased mechanical pulling-force by enhancing cell–cell adhesion. In the following paragraphs, we will summarize the current insights and the most recent findings on the molecular events that underlie such mechanotransduction responses at endothelial cell–cell junctions.

### Cytoskeletal-dependent remodeling of VE-cadherin-based cell–cell junctions

**Fig. 1:**
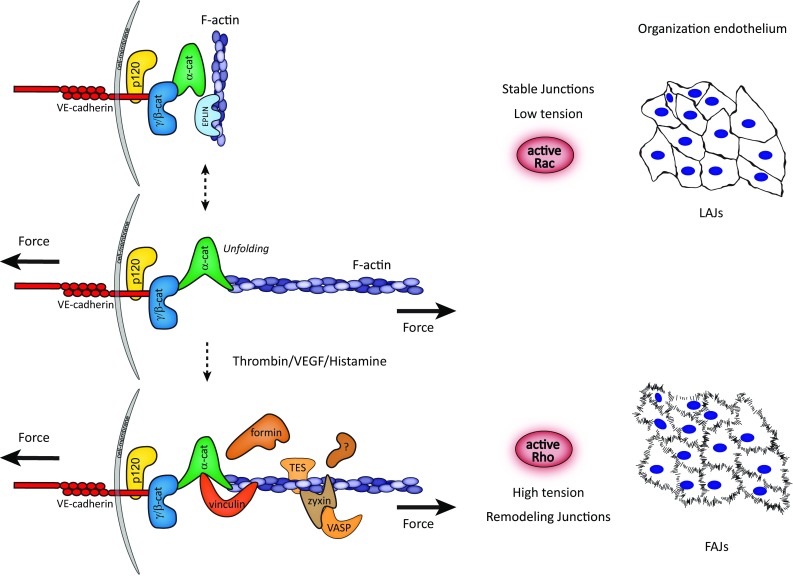
Mechano-transduction events during remodeling of endothelial adherens junctions. A model depicts remodeling phases of the VE-cadherin complex in response to pulling forces. In stable monolayers, cell–cell adhesions are organized as continuous linear adherens junctions (LAJs). This conformation is promoted by Rac-driven cell protrusions which lower tension on the VE-cadherin complex and allow the formation of cortical F-actin bundles. Together, these events support cell–cell adhesions and enhance barrier function. Cytoskeletal dynamic is responsible for a kinetic system of actin-bound and -unbound states of the VE-cadherin complex, in which pulling forces from the actomyosin cytoskeleton stabilize a direct interaction of F-actin with unfolded junctional α-catenin. Permeability agonists that stimulate Rho-mediated actomyosin contractility induce the formation of tensile radial F-actin bundles, which pull on the VE-cadherin complex. High pulling-derived tension destabilizes cell–cell adhesions, which adopt a discontinuous focal adherens junction (FAJ) organization, and induce endothelial permeability. Tension-induced binding of vinculin to α-catenin marks the formation of FAJs, and is responsible to protect these junctions from opening too far. Simultaneously, several actin remodeling proteins, including zyxin, VASP, and TES, are recruited to FAJs upon cytoskeletal-derived pulling

Formation of stable adherens junctions requires coupling of the VE-cadherin intracellular domain via a cytoplasmic protein complex to the actin cytoskeleton. This complex is also critical for most junctional mechanotransduction events. Moreover, actin dynamics tightly control the assembly and disassembly of VE-cadherin-based junctions [19, 20]. In cultured endothelial cells, the formation and stabilization of cell–cell adhesions are promoted by actin-protrusive structures that locate at or near the junctions [21–23]. In mature stabilized junctions, VE-cadherin is linearly or continuously organized between cells and supported by parallel running cortical actin bundles [23, 24]. The transition of stable into cytoskeletal-dependent remodeling junctions is mediated by actomyosin contractions that generate pulling tension on the junctions [25, 26]. Such remodeling induced by cytoskeletal-pulling forces results in the formation of a discontinuous junction type, connected to perpendicular tensile actin bundles, which we call Focal Adherens Junction (FAJ; Fig. 1) [25]. The switching between stable and remodeling junctions is tightly controlled by the localized activation of small GTPases that modulate cytoskeletal dynamics [27]. Local activation of the GTPase Rac supports junction stabilization (linear junctions), which corresponds with a release of tension from VE-cadherin [21, 28]. Vice versa, the activation of the GTPase Rho increases actomyosin-mediated pulling forces on endothelial junctions and promotes the formation of FAJs (Fig. 1) [18, 25]. It seems likely that for efficient barrier function of endothelial monolayers, both the protrusive and contractile activities of the actin cytoskeleton are important, as these actin dynamics enable individual cells to respond to, and resist the pushing and pulling of their neighbouring cells in monolayer tissue [22, 29, 30].

How such force-dependent junction remodeling relates to the function of vascular endothelial junctions in vivo is less clear, although recent advances have been made in studies that visualized the remodeling of endothelial cell–cell contacts and the actin cytoskeleton during angiogenesis and anastomosis in transgenic models [31, 32]. Importantly, temporal control of the interaction between F-actin and the VE-cadherin complex turns out to be crucial for agonist-evoked vascular permeability and leukocyte extravasation in adult mice [33]. In addition, the distinct organizations of endothelial junctions are recognizable within human blood vessels [34], which indicates that force-dependent adherens junction conformations observed in cultured monolayers relate well to remodeling of endothelium in vasculature.

### VE-cadherin-dependent mechanotransduction: switching α-catenin conformation

In a simplified model of adherens junctions, the connection between classical cadherins and the F-actin cytoskeleton is made by a core protein structure that consists of the cadherin/β-catenin/α-catenin chain, which connects to actin filaments (Fig. 1). Of note, in endothelial cells, β-catenin may be replaced in this chain by its homologue γ-catenin (plakoglobin) [35, 36]. In the past years, the role of α-catenin in bridging the junction and the actin cytoskeleton has been extensively explored, which was triggered by biochemical studies showing that α-catenin does not bind β-catenin and actin simultaneously in solution [37, 38]. Current models point towards the dynamic and allosteric regulation of α-catenin in response to mechanical forces in cells, and it becomes clear that α-catenin is a key mechanosensor interacting with proteins, including actin, in a force-dependent manner (Fig. 1) [39–42]. Monomeric α-catenin binds strongly to the cadherin/β-catenin complex, but weakly to F-actin and the affinity of α-catenin for F-actin decreases even further upon binding to cadherin/β-catenin [42]. Intriguingly, using an optical trap-based assay to measure the lifetime of the interaction between the cadherin core complex to actin fibers, Buckley and colleagues discovered that the exertion of tensional force to a reconstituted cadherin/catenin complex highly increases binding of α-catenin to F-actin by forming strong long-lived bonds [42]. This work has put emphasis on a kinetic model of actin-bound and -unbound states of the cadherin complex, in which optimal mechanical tension stabilizes the direct interaction of F-actin to the cadherin complex. Such a model is also supported by studies using recently developed FRET probes of α-catenin, which revealed the conformational changes of the protein as a function of altered tension at cell–cell junctions [43]. α-catenin lacking the β-catenin or F-actin-binding domains do not display force-dependent changes in conformation, indicating that both protein interactions are necessary for α-catenin to function as a mechanotransducer.

### Force-induced α-catenin–vinculin interaction

Cytoskeletal pulling at the cadherin complex not only alters its F-actin-binding affinity in a direct manner, but indirectly it might also stabilize the F-actin connection through the recruitment of vinculin. Junctional recruitment of vinculin occurs via α-catenin and depends on actomyosin-based contractile forces (Fig. 1) [40, 41]. Mechanical pulling experiments using magnetic tweezers on single α-catenin molecules demonstrate that within the physiological range of cytoskeletal-pulling forces, α-catenin unfolds and exposes a protein-binding domain for vinculin. Recruited vinculin, in turn, stabilizes the unfolded conformation of α-catenin [44]. Interestingly, the FRET-based studies of Kim and colleagues further indicate that the alterations of α-catenin conformation precede the recruitment of vinculin, and suggest that vinculin is not necessary for force-induced regulation of α-catenin per se [43]. Nevertheless, vinculin binding stabilizes α-catenin in its open conformation and after force release; vinculin slows down the process of refolding of α-catenin [44], further supporting earlier findings of crosstalk between the two proteins upon binding [39, 45, 46]. It is still unclear whether the conformation and binding properties of vinculin itself (or VE-cadherin and β-catenin) alter at cell–cell junctions under tension. Possibly, FRET-based sensors developed for vinculin [47] and VE-cadherin [48] might reveal new insights that address this issue.

In endothelial cells, and many other cell types, vinculin recruitment to cell–cell junctions demarcates force-dependent remodeling. Vinculin is absent from Rac-induced linear adherens junctions, which experience low levels of tension across VE-cadherin [28]. By contrast, junctions that are remodeled by increased pulling forces (FAJs) specifically recruit vinculin [18, 25]. Furthermore, VE-cadherin directly serves as the mechanotransducing receptor that is responsible for vinculin recruitment, and F-actin accumulation, in response to mechanical forces derived from magnetic twisting cytometry with VE-cadherin-coated beads [49]. Similar experiments that exert force on PECAM-1-based adhesions did not trigger vinculin recruitment or F-actin accumulation. This indicates that junctional recruitment of vinculin occurs specifically via the mechanical stimulation of VE-cadherin. Specific perturbation of the force-dependent α-catenin–vinculin interaction further reveals that vinculin functions as a strengthener of cell–cell adhesion and barrier formation [25, 50]. The physiological consequence of vinculin recruitment to endothelial junctions still remains to be investigated. We do know that endothelial permeability agonists promote the formation of FAJs (Fig. 1) and recruitment of vinculin to these junctions protects them from opening too far during their permeability-response [25]. This suggests that VE-cadherin-dependent mechanotransduction plays a role in limiting vascular leakage during inflammatory responses.

### Other force-dependent molecular events at cell–cell junctions

Thus far, we discussed mechanotransduction concentrated around regulation of the α-catenin–vinculin interaction and the connection of F-actin to the VE-cadherin complex. In addition to direct conformational changes induced by tension, force-modulated phosphorylation of α-catenin and vinculin further contributes to their role in mechanotransduction [51–54]. Besides local vinculin recruitment and reinforcement of adhesion, VE-cadherin-mediated mechanotransduction also induces global signals that confer cell stiffening, remodeling of distant integrin-based focal adhesions, and adherens junctions [49]. Similar responses are observed when applying force on PECAM-1-based adhesions [55]. These findings emphasize that VE-cadherin-mechanotransduction occurs within an integrated, mechanosensitive network that regulates both local remodeling at the site of force application and the global integrity of endothelial tissue.

Moreover, there are additional proteins present at the cadherin-F-actin interface, and it is likely that other actin-binding proteins, which interact with α-catenin, may contribute or respond to VE-cadherin-dependent mechanotransduction. This might involve proteins like epithelial protein lost in neoplasm (EPLIN), the tight junction protein ZO-1, afadin, α-actinin, and formin-1 [13]. Moreover, the actin regulatory proteins VASP, zyxin, and TES are specifically recruited to force-dependent FAJs in endothelial cells in similar kinetics as vinculin does, but their recruitment occurs clearly independent of the previously described α-catenin–vinculin mechano-interaction [56]. Instead, recruitment of zyxin and TES to FAJs requires their functional LIM domains [56], protein interaction domains which recognize cytoskeletal remodeling in response to force [57]. Also the actin bundling protein fascin is recruited to nascent endothelial AJs that are very reminiscent of force-induced FAJs [58]. Conversely, EPLIN, another LIM domain protein that interacts with α-catenin and F-actin, is recruited to endothelial adherens junctions [59]. However, the junctional recruitment of EPLIN to linear adherens junctions occurs in particular after the release of tension, and EPLIN is excluded from vinculin-positive FAJs [60], which points towards an alternative force-dependent-event. We speculate that the presence of these various actin regulatory proteins indicates that actin polymerization and bundling are of key importance for force-dependent regulation of endothelial AJs.

Besides through VE-cadherin, cell–cell junctions are formed in conjunction with various other receptors, and it is very likely that multiple receptors take part in force-induced junction remodeling. For instance, the presence of the tight junctional protein ZO-1 in endothelial cells is responsible for myosin II activation near cell–cell junctions [61]. By measuring a VE-cadherin-based FRET sensor, the authors further show that the depletion of ZO-1 results in a significant loss of tension from the VE-cadherin complex. These findings implicate the existence of a mechanism, by which tight junctions regulate VE-cadherin-dependent mechanotransduction. Moreover, the presence of another transmembrane adhesion receptor EMMPRIN (extracellular matrix metalloproteinase inducer) at endothelial junctions is important for myosin II activity during the maturation of VE-cadherin-based junctions [62].

## Flow mechanosensing: roles of PECAM-1 and VE-cadherin

**Fig. 2:**
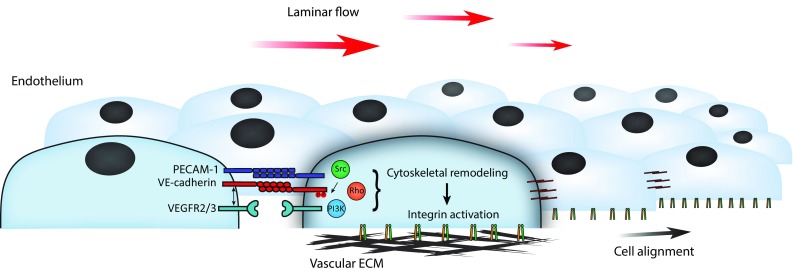
Mechano-transduction events during endothelial sensing of laminar flow. Shear forces derived from the bloodstream promote endothelial signaling. This occurs via a mechanotransduction complex consisting of the junctional adhesion proteins PECAM-1 and VE-cadherin in conjunction with activation of VEGF receptors. Subsequently, activation of signaling pathways controlled by Src, Rho, and PI3K mediates cytoskeletal remodeling and activation of basal integrins which support alignment of the endothelial cells in the direction of flow

The transmembrane adhesion receptor PECAM-1 mediates homotypic adhesion between endothelial cells and contributes to maintenance of the endothelial barrier [63]. Moreover, PECAM-1 is a key mechanotransducer that converts shear forces derived from laminar blood flow into endothelial cell alignment in the direction of flow [17]. After application of apical flow, activation of integrins on the basal surface induces cytoskeletal-mediated cell alignment. Both PECAM-1 and VE-cadherin-based adhesions are crucial for flow-induced integrin activation (Fig. 2) [17]. This mechano-response is likely dependent on direct force exerted on PECAM-1, as local application of tensional force on PECAM-1-adherent beads elicits global cytoskeletal stiffening, which, in turn, underlies remodeling of the basal integrin-based adhesions [55]. Possibly, direct application of flow-dependent force on VE-cadherin-based adhesions further enhances this response. However, studies with FRET-based tension sensors for PECAM-1 and VE-cadherin indicate that flow promotes tension on PECAM-1, within the range of pN force, whereas tension on VE-cadherin, in fact, lowers [48]. Both vimentin and actomyosin activity are crucial for the flow-induced increased tension on PECAM-1 as well as for cell alignment [48]. Endothelial signals induced by flow, that may explain basal responses to apical applied forces on cell–cell junctions, include activation of Rho, PI3K, and Src family kinase (Fig. 2) [17, 49, 55, 64]. Activation of PI3K after flow is triggered by transactivated vascular endothelial growth factor receptors (VEGFR2 and VEGFR3), and it was recently shown that VE-cadherin interacts via its transmembrane domain with these receptors and thereby supports their downstream signaling [65]. In summary, current models indicate that the VE-cadherin complex is a direct mechanotransducer during cell–cell junction remodeling upon cytoskeletal-pulling forces (see earlier paragraphs). Conversely, during flow sensing, VE-cadherin seems to function rather as an adaptor for VEGFR signaling towards remodeling of integrins. Yet, Src-dependent phosphorylation of the cytoplasmic tail of VE-cadherin at Y658 and Y685 is strongly dependent on the speed of flow [66]. Because blood flow rates are distinct in arteries versus veins [67], this may explain why phosphorylation of these specific residues occurs preferentially in veins and not in arteries [66, 68]. Until now, it is unclear whether phosphorylation of VE-cadherin is actively involved in flow-induced mechanotransduction. Of interest, the small GTPase Rap1, which is strongly implicated in endothelial cell–cell junction stabilization and barrier formation [69], is required for functioning of the PECAM-1/VE-cadherin/VEGFR complex in flow sensing [70]. This adds another signaling route to this mechanotransduction pathway. Finally, it is already long known that shear stress induces currents across the plasma membrane of endothelial cells, for which mechanosensitive ion channels are responsible [71–73]. Of these mechano-channels, the endothelial-expressed transient receptor potential cation channel subfamily V member 4 (TRPV4) has been recently reported to be presented at higher levels in response to flow [74], and to interact with β-catenin at cell–cell junctions [75, 76]. This hints at a potential role for cell–cell junctions in Ca^2+^-dependent signaling during adaptation to flow-derived forces.

## Intracellular mechanotransduction

Mechanical stimuli, initially sensed by transmembrane (adhesion) complexes, propagate throughout the cell via direct mechanotransduction and via force-induced biochemical signaling [77, 78]. Eventually, these events lead to cytoskeletal remodeling (i.e., actomyosin-mediated stiffening), endothelial alignment, and nuclear adaptation [79]. Rho GTPases control the actomyosin cytoskeleton and take a central role in endothelial mechano-signaling [3, 80, 81]. Permeability agonists, such as thrombin and histamine, induce the formation of force-dependent FAJs via activation of Rho [18, 25, 82–84]. In turn, Rho signaling via its effector Rock assures resilience of the cytoskeleton to withstand external forces [85], which likely protects endothelial integrity during inflammatory responses [3, 18, 86]. In addition, Rho–Rock signaling is crucial for the stiffening mechano-responses that are triggered by direct force applied on PECAM-1- and VE-cadherin-based adhesions [48, 49, 55]. Key identified endothelial Rho activators (so-called GEFs) that are responsible for adhesion-mediated mechanotransduction are LARG and GEF-H1 [87, 88]. However, it is unknown which GEFs mediate mechanotransduction from endothelial adherens junctions. A recent functional screen for GEFs in endothelial reorientation after mechanical substrate stretching (which may relate to physiological relaxation and contraction of the vessel wall) suggests a role for the GEF Solo in junction-dependent mechanotransduction [89]. Of note, ten additional GEFs were identified to be involved in this mechanically induced response, and it is still unclear whether Solo is activated downstream from VE-cadherin, PECAM-1, or alternative endothelial mechanoreceptors.

Intriguingly, cytoskeletal stiffening enhances transmission from extracellular forces towards mechano-responses within the nucleus [55, 77, 85]. Changes in shear stress regulate endothelial gene expression and there is a growing list of flow-sensitive miRNA’s of which the miRNA’s-19a, -21, -92a, -143, -145 and -712 target vascular permeability and inflammatory pathways [90–95] It seems likely that for these processes, the nucleus connects to the stiffening cytoskeleton. The LINC (Linker of Nucleoskeleton and Cytoskeleton) protein complex governs this function. It contains KASH (Klarsicht, Anc-1, Syne Homology)-domain proteins, such as nesprins, that span the outer nuclear membrane and interact with the cytoskeleton. Furthermore, the complex includes SUN and emerin proteins that span the inner nuclear membrane [96, 97], which, in turn, connect to the nucleoskeleton via lamins and regulate chromatin dynamics and gene expression [98]. Another recently discovered mechano-signaling pathway that biochemically couples extracellular mechanical stimuli to nuclear responses comprises the activity of Yes-associated protein (YAP) and transcriptional co-activator with PDZ-binding motif (TAZ) proteins. Being part of the conserved Hippo pathway, these proteins are responsible for cell–cell contact inhibition and inhibition of cell proliferation to regulate tissue size [99]. Independent from activation via the Hippo pathway, YAP/TAZ activation is regulated by cytoskeletal contractility and Rho GTPase activity [100]. The activity of these transcriptional regulators is controlled by numerous mechanical stimuli, including extracellular matrix (ECM) stiffness, cell geometry, cell–cell contact, and shear stress [101]. Endothelial cells adhering to flexible ECM show cytoplasmic YAP/TAZ localization, whereas in intermediate or high stiffness environments, the proteins locate within the nucleus [100]. This relocalization is linked to the activity of YAP/TAZ and can also be observed upon mechanical cell stretching, altered cell-polarity, or cell–cell adhesion. For endothelial cells, the YAP/TAZ mechanotransduction pathway is important for geometry determined cell survival [100]. To date, the precise mechanisms by which YAP/TAZ sense mechanical stimuli, and if and how they relate to remodeling of cell–cell junctions, are not fully clear. However, the function and junctional recruitment of YAP tightly depend on VE-cadherin-based adhesion and are modulated by the tension-raising permeability agonist thrombin [102]. Moreover, the actin remodeling protein EGF receptor kinase substrate 8 (EPS8) has recently been shown to regulate the interaction of YAP with the VE-cadherin complex and to control endothelial permeability in vivo [103]. Recruitment of EPS8 to cell–cell junctions occurs via binding with α-catenin and is particularly prominent during junction remodeling in subconfluent endothelial monolayers. EPS8 binding competes with the interaction of YAP to the VE-cadherin complex and regulates VE-cadherin turnover. Upon adherens junction maturation, EPS8 dissociates from the VE-cadherin complex, and PI3K-mediated phosphorylation of YAP promotes its recruitment to cell–cell junctions and renders YAP transcriptionally inactive [103]. As mentioned before, force applied on cell–cell junctions activates PI3K, and therefore, this molecular event may provide a link between junctional mechanotransduction and transcription in maintenance of endothelial integrity. Taken together, control of endothelial YAP/TAZ is a relatively new field of expertise, and clearly, more in-depth investigations will be needed to fully unravel the importance of the crosstalk between junctional and nuclear mechanotransduction.

## Junction remodeling in angiogenesis

Sprouting angiogenesis, a physiological remodeling process, in which new blood vessels emerge from existing vasculature [104, 105], is tightly dependent on modulation of cell–cell junctions and various mechanical forces [9, 106]. At the onset of angiogenesis, remodeling of endothelial cell–cell adhesions is required to allow sprout formation, whereas at later stages, when new sprouts are established and lumens form, cell–cell adhesions tighten and establish vessel integrity. As crucial cell–cell adhesion receptor, VE-cadherin plays a key role in these processes: endothelial cells expressing lower levels of VE-cadherin [107], or lacking functional VE-cadherin [32, 108] fail to correctly organize cell–cell junctions in forming sprouts. As a consequence, angiogenesis is perturbed due to a weakened interaction of tip cells with following stalk cells or due to sprouts failing to establish a connection to the pre-existing vasculature.

Based on a combination of computational modeling and live imaging of angiogenesis, Bentley and colleagues proposed that differential junctional adhesive strength throughout the vasculature, via changes in VE-cadherin mobility, allows for endothelial cell rearrangement and underlies the formation of angiogenic sprouts [109]. It is not completely clear when forces at junctions, or direct VE-cadherin-dependent mechanotransduction, are at play in sprouting angiogenesis. However, it is evident that during the different phases of angiogenesis, remodeling adherens junctions between tip and stalk cells appear, which are reminiscent of the force-dependent FAJs observed in endothelial cultures [109]. The actin cytoskeleton is highly dynamic at endothelial junctions during angiogenesis [31, 110]. Moreover, endothelial actomyosin contractility, which generates cytoskeletal force, regulates the distribution of VE-cadherin at cell–cell adhesions [111]. Conversely, optimal ECM rigidity controls sprout formation and vascular network connectivity [112], likely caused by feedback mechanisms derived from the ECM that determines collective behaviour of endothelial cells [113], and due to altered responsiveness to angiogenic growth factors, such as VEGF [114]. The importance of crosstalk between cell–cell junctions, the cytoskeleton, and interactions with the ECM is further supported by the finding that endothelial β1 integrins control angiogenic sprouting via the actomyosin-dependent distribution of VE-cadherin and stabilizing cell–cell junctions in maturating vessels [115]. During lumen formation, the scaffold protein AmotL2 is needed for proper connection of VE-cadherin to the F-actin cytoskeleton [116]. Because AmotL2 is also required for actomyosin-dependent forces at endothelial junctions, this finding suggests that VE-cadherin mechanotransduction, via its coupling to the cytoskeleton, underlies lumen formation in newly formed vessels. During the process of collective migration, for example, in elongating sprouts, endothelial cell–cell junctions experience changes in mechanical tension. During their remodeling these junctions are stabilized by local F-actin assembly, for which the Rho effector formin-like 3 (FMNL3) is crucial [117, 118]. Moreover, inhibition of formin activity readily converts stable LAJs into remodeling FAJ in vitro. *In vivo* inhibition of formin activity perturbed lumen formation [118, 119]. Interestingly, the related protein formin-1 interacts with α-catenin [120], within the same domain, where the force-induced interaction of α-catenin with vinculin occurs [13]. We speculate that junctional recruitment of FMNL3 could be part of a VE-cadherin-dependent mechanotransduction in angiogenesis.

In addition to mechanical forces induced by collective cell migration, mechanical forces derived from blood flow will further contribute to control angiogenesis [121]. For instance, once the level of increasing shear stress reaches a certain threshold, the formation of sprouts is promoted [122]. Surprisingly, no prominent role for VE-cadherin-based junctions was found in this mechano-response, emphasizing a role for alternative mechanotransduction mechanisms in angiogenesis.

In lymphatic vasculature, a junctional remodeling process is observed in the collecting lymphatics, where PECAM-1- and VE-cadherin-based junctions are separated at a distinct button-like structure that allows fluid entry from tissue [123, 124]. At those button-like junctions, the adherens junctions specifically adopt an interrupted conformation, comparable to the organization of FAJs in vascular endothelium. The (lymph)angiogenic growth factor angiopoietin-2 induces the formation of button-like junctions during the development of collecting lymphatics and triggers phosphorylation of VE-cadherin at Y685, the latter being a mechanotransduction response induced by flow-derived forces [66]. Another event which takes place in collecting lymphatics is triggered by disturbed flow, which activates the transcription factor FOXC2. The presence of FOXC2 is responsible for recruitment of YAP/TAZ to lymphatic endothelial junctions and stabilizes endothelial integrity in disturbed flow conditions, thereby supporting formation of functional collecting lymphatics [125].

Taken together, tight interplay between junctional remodeling and mechanical forces occurs during (lymph)angiogenesis. We expect that novel developments in in vivo imaging models, using transgenic zebrafish or mouse models, will further establish the importance of mechanotransduction events at the distinct steps of the angiogenic cascade.

## Mechanotransduction in vascular stiffness-related disease

Blood vessel stiffening is an important cause of leakage and inflammation in age-related vascular diseases, including hypertension and atherosclerosis. For example, stiffness of the aorta increases aortic pulse pressure, pressure wave velocity, leading to hypertension, and is a strong predictor of cardiovascular morbidity and mortality [126, 127]. In addition, vascular stiffening associates with acute respiratory distress syndrome and vascular injury. Arteries stiffen as a result of structural changes in the ECM of the blood vessel wall during aging [1, 2]. ECM turnover and changes in its composition (mainly collagens, fibronectin, elastin and calcium deposits) determine the level of vascular stiffening. During age-related vessel stiffening, deposition of various collagen types increases, not only at the subendothelial level, but also in the intima and media layers of the vasculature [128, 129]. Accumulation of advanced glycation end-products (AGEs) reinforces this process by increasing the crosslinking of collagen [130]. Elastin levels decrease in the vessel wall during aging, which is considered an irreversible process, underlying a large part of the stiffening process [131]. Besides such alterations in the ECM, changes in the activity and structure of vascular smooth muscle cells with aging promote vessel stiffness [132]. Even though the actual stiffness of the vascular wall of carotid arteries denuded from endothelium is similar as in intact arteries [133], a role for endothelial cells in stiffening of the vascular wall is expected to occur via reduced production of nitric oxide, which promotes vasoconstriction via vascular smooth muscle cell activation [134]. In addition, disturbances in blood flow, e.g., at arterial bifurcations or at locations of vascular damage trigger local stiffening and the formation of atherosclerotic plaques [135]. Of note, the extent of forces induced by ECM stiffening, and exerted on endothelial adhesion receptors, is orders of magnitudes higher than those derived from blood flow [136]. Stiffening of the subendothelial matrix from 2.5 kPa (a condition mimicking young arteries) to 10 kPa (a condition comparable to arteries of older individuals) already has major impact on the atheroprotective role of fluid flow [137]. Endothelium grown on top of 2.5 kPa conditions promotes tightening of endothelial cell–cell junctions, lowering of RhoA GTPase activation, and production of endothelial nitric oxide in response to arterial flow [137]. In addition, the type and magnitude of shear stress derived from flow have major impact on flow-initiated endothelial mechanotransduction responses, including augmented intracellular forces [138]. Future studies are expected to unravel the intriguing crosstalk of mechanotransduction involved in simultaneous sensing of forces from ECM and flow.

The eventual leakage and inflammatory response in stiffening vessels are concluded at the level of the endothelium. As discussed earlier, there is tight crosstalk between cell–matrix and cell–cell adhesion (reviewed in detail [139, 140]). Integrin-mediated mechanotransduction translates forces from the ECM to actomyosin-mediated pulling [141], which, in turn, regulates endothelial cell–cell adhesions [18, 25, 142]. Pathophysiological stiffening of the vessel wall perturbs this mechanotransduction response and increases monolayer permeability, leukocyte transmigration, and drives cardiovascular disease [83, 143, 144]. The importance of this pathway is underscored by the finding that in vivo deficiency of non-muscle myosin light-chain kinase, an important activator of actomyosin contraction, attenuates endothelial permeability and atherosclerosis [145]. Furthermore, in mice, where endogenous VE-cadherin is replaced by a VE-cadherin-α-catenin fusion protein (which tightens junctions to F-actin), inflammatory-induced vascular leakage and leukocyte transmigration are strongly reduced [33], pointing to a potential role of junctional mechanotransduction as therapeutic target in inflammation. As discussed earlier, endothelial mechanotransduction includes signals that promote permeability, but also signals that protect against permeability. It is fair to assume that pathological ECM stiffness perturbs the balance between permeability protective and promoting mechanisms. Moreover, flow-induced mechano-responses depend on the stiffness of the vascular ECM [137], which will be important for structural vessel remodeling upon strong changes in flow (i.e., after bypass surgery or in arteriovenous fistulas). Deformation of the endothelium during these processes is translated to cytoskeletal and junctional adaptation through, for example, phosphorylation of adhesion proteins [146]. Altogether, the discovery of integrated mechanotransduction responses, controlling endothelial cell–cell junctions, and barrier function opens up possibilities to restore this balance and to reduce stiffness-associated vascular disease. Potentially, advances in endothelial proteomics will identify those junctional mechanotransduction events that may serve as targets to block permeability and inflammation in stiffened arteries. Recent proteomic studies in non-endothelial cells have identified novel networks of proteins that associate with cell–cell junctions [147, 148]. By analyzing the identified proteins from those mass spectrometry studies in the Online Mendelian Inheritance in Man (OMIM) database, we find associations with cardiovascular disease (Table 1). Most of these associations show a link with cardiac phenotypes, which could relate to the close crosstalk between forces at the vascular wall, hypertension, and cardiac remodeling [149, 150]. The current challenge is to find (novel) key events that may serve as therapeutic targets to prevent vessel leakage and inflammation.

**Table 1:** Genetic association of junction proteins with inflammation and cardiovascular disease

Gene	Protein	Disease
ADAM17	A disintegrin and metalloproteinase domain 17	Neonatal inflammatory skin and bowel disease
ADD1	Adducin 1	Hypertension
BAG3	Bcl2-associated athanogene 3	Dilated cardiomyopathy, myofibrillar myopathy
BMPR2	Bone morphogenetic protein receptor, type II	Familial primary pulmonary hypertension, Pulmonary venoocclusive disease
CD2AP	CD2-associated protein	Focal segmental glomerulosclerosis
CTNNA3	α-T-catenin	Arrhythmogenic right ventricular dysplasia
DSC2	Desmocollin 2	Arrhythmogenic right ventricular dysplasia
DSG2	Desmoglein 2	Arrhythmogenic right ventricular dysplasia
FLNA	Filamin-A	X-linked cardiac valvular dysplasia
JAG1	Jagged 1	Congenital heart defects, Alagille syndrome
JUP	Plakoglobin, γ-catenin	Arrhythmogenic right ventricular dysplasia
NOTCH1	Notch homolog 1 (*Drosophila*)	Aortic valve disease
NOTCH3	Notch homolog 3 (*Drosophila*)	Cerebral arteriopathy
Nup155	Nucleoporin, 155-kDa	Atrial fibrillation
PKP2	Plakophilin 2	Arrhythmogenic right ventricular dysplasia
PS1	Prenesilin 1	Dilated cardiomyopathy
TMPO	Thymopoietin	Dilated cardiomyopathy
TTN	Titin	Dilated cardiomyopathy, familial hypertrophic cardiomyopathy
VCL	Vinculin	Sporadic and familial dilated cardiomyopathy; Hypertrophic cardiomyopathy
